# Circulating Betatrophin in Patients with Type 2 Diabetes: A Meta-Analysis

**DOI:** 10.1155/2016/6194750

**Published:** 2015-12-01

**Authors:** Sheyu Li, Dan Liu, Ling Li, Yun Li, Qianrui Li, Zhenmei An, Xin Sun, Haoming Tian

**Affiliations:** ^1^Department of Endocrinology and Metabolism, West China Hospital, Sichuan University, Chengdu, Sichuan 610041, China; ^2^Chinese Evidence-Based Medicine Center, West China Hospital, Sichuan University, Chengdu, Sichuan 610041, China; ^3^Department of Endocrinology and Metabolism, The Third People's Hospital of Chengdu, Chengdu, Sichuan 610031, China; ^4^Department of Endocrinology and Metabolism, West China Hospital, Sichuan University, No. 37 Guoxue Road, Chengdu, Sichuan 610041, China

## Abstract

*Objective*. To investigate the association between circulating betatrophin level and type 2 diabetes mellitus (T2DM) in human.* Methods*. A comprehensive literature search was performed in PubMed and Embase databases to identify eligible studies assessing the circulating levels of betatrophin in both T2DM patients and nondiabetic adults.* Results*. A total of nine eligible studies with twelve comparisons were included for the final meta-analysis. Circulating betatrophin levels in T2DM patients were higher than those in the nondiabetic controls (random-effect SMD 0.53; 95% CI 0.13 to 0.94; *P* = 0.010). In the subgroup of nonobese population but not the obese population, the overall betatrophin level in T2DM patients was much higher than that in the nondiabetic controls (nonobese: random-effect SMD, 0.82; 95% CI 0.42 to 1.21; *P* < 0.001; obese: random-effect SMD, −0.39; 95% CI, −0.95 to 0.18; *P* = 0.18). Metaregression indicated that body mass index of T2DM patients was associated with mean difference of betatrophin level between T2DM and nondiabetic adults (slope, −578.8; *t* = −2.7; *P* = 0.02).* Conclusion.* Based on the findings of our meta-analysis, circulating betatrophin level of T2DM patients is higher than that of nondiabetic adults in the nonobese population, but not in the obese population.

## 1. Introduction

Betatrophin, also known as lipasin, angiopoietin-like protein (ANGPTL8), refeeding induced fat and liver (RIFL), and chromosome 19 open reading frame 80 (TD26), is a newly identified circulating protein predominantly secreted from the liver in humans [1–5]. It has been well established that betatrophin is a novel regulator of lipid metabolism in both human and rodents [1–4, 6, 7]. Recent studies indicated that high betatrophin level was associated with islet *β*-cell proliferation in mice [8], but knockout of betatrophin failed to impair the glucose profiles of mice [9].

In human being, observational studies indicated that circulating betatrophin was associated with a variety of health conditions, including type 2 diabetes (T2DM) [10–16]. However, the correlation between betatrophin level and T2DM remained controversial, with studies indicating both positive and negative results [17]. The potential causes of these conflicted results were poorly described. In the current meta-analysis, we aimed to investigate the association between circulating betatrophin level and T2DM in human and to explore the possible causes of heterogeneity.

## 2. Methods

### 2.1. Search Strategy

The meta-analysis was conducted following Meta-Analyses of Observational Studies (MOOSE) guideline (checklist shown in Supplementary Table 1 in Supplementary Material available online at http://dx.doi.org/10.1155/2016/6194750). Using the terms betatrophin, ANGPTL8, lipasin, C19ORF80, TD26, and RIFL, we comprehensively searched the PubMed and Embase databases up to March 27, 2015, for studies assessing the circulating levels of betatrophin in both T2DM and nondiabetic adults. Searches were limited to studies published in English language. We also reviewed the reference lists of included papers for potentially relevant publications.

### 2.2. Study Selection

Studies were enrolled if they were (1) case-control studies comparing circulating betatrophin levels in T2DM patients and nondiabetic controls or (2) observational cohort studies reporting T2DM prevalence in different patients with diverse baseline circulating betatrophin levels. Studies with insufficient data and meeting abstracts were excluded.

### 2.3. Data Extraction and Quality Assessment

Two reviewers (S. L. and D. L.) independently reviewed all searched studies and extracted data using a predefined form. The following information of each study was recorded: first author, country of the study, participant recruitment, sample size, betatrophin measurement, age, gender, and other baseline parameters, like body mass index (BMI), lipid profiles, and so forth. The methodological quality of each included study was assessed by two reviewers independently using the Newcastle-Ottawa Quality Assessment Scale (NOS) [18]. The scale consists of nine items that cover three categories: participant selection (four items), comparability (two items), and exposure (three items). A study can be given a maximum of one star for each numbered item within the* Selection* and* Exposure* categories and a maximum of two stars for* Comparability*. Discrepancies between the two reviewers were resolved by discussion with a third reviewer (H. T.).

### 2.4. Statistical Analysis

If the data of an included study were not provided as mean ± standard deviation (SD) with sample size larger than 25 [10–16, 20, 22], we transformed standard error of mean (SEM) or the interquartile range (IQR) to SD by the following formula (a, b) and use median to estimate the mean:

(a) SD = SEM *∗* $\sqrt{n}$,
(b) SD = IQR/1.35.

Standard mean difference (SMD) and 95% confidence interval (95% CI) were used to assess the differences in circulating betatrophin levels between groups between studies. Cochran's *Q* statistics and the *I* ^2^ statistics were used to assess the heterogeneity between studies. A random-effect model was used in the meta-analysis due to nonnegligible heterogeneity between studies. We also did subgroup analyses and metaregression to explore the potential source of heterogeneity if heterogeneity across studies was statistically significant. To investigate the publication bias, we performed Begg's test with a level of significance being *P* < 0.05. All analyses were carried out using Stata statistical software version 12.0 (StataCorp, College Station, TX, USA).

## 3. Results

### 3.1. Literature Search

**Figure 1:**
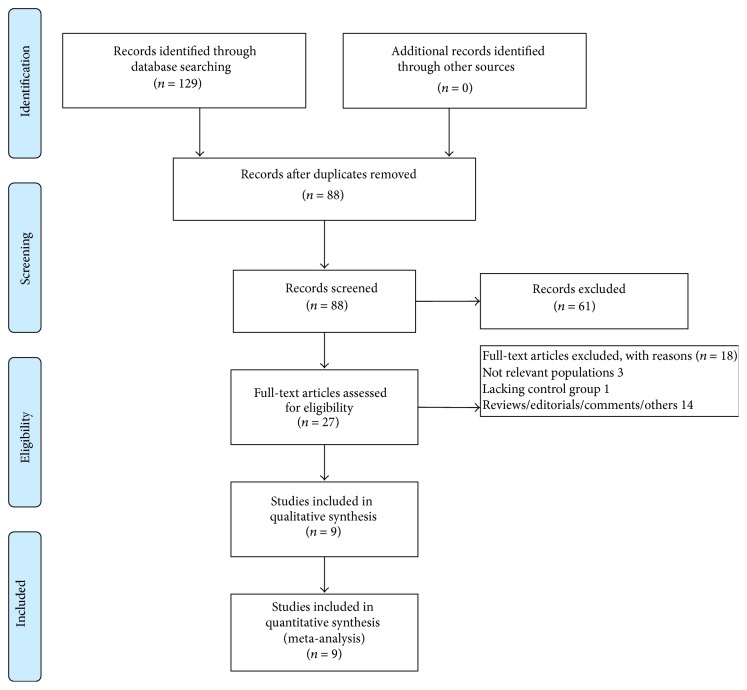
Flow diagram of study recruiting.

As shown in Figure 1, we identified 129 relevant records through searching the PubMed and Embase databases and excluded 102 of them after deduplication and title/abstract screening. After a full text review, nine studies including twelve comparisons were finally included for meta-analysis (rationale and list for each excluded paper were shown in Figure 1 and Supplementary Information, resp.) [10–16, 20, 22].

### 3.2. Study Characteristics and Quality Assessment

All included studies were designed as case-control studies including 417 T2DM patients and 477 nondiabetic controls. The characteristics of them were shown in Tables 1 and 2. The NOS of each study ranged from 4 to 8 (Table 1 and detailed scoring in Supplementary Table 2).

All the nine studies with twelve comparisons recruited patients with T2DM, of which three recruited patients with newly diagnosed T2DM [13–15], one recruited patients undergoing chronic hemodialysis [12], and three enrolled obese T2DM patients [13, 20, 22]. Five of the twelve comparisons recruited patients with ongoing antidiabetic treatment [10, 12, 16, 20], and three of them [10, 16, 20] reported the prescription information of the studied cases (shown in Supplementary Table 3). Circulating betatrophin levels in all included studies were examined after overnight fasting. Two studies including three comparisons used the enzyme-linked immunosorbent assay (ELISA) kit provided by Phoenix Pharmaceuticals (Catalogue number EK-051-55; Burlingame, CA, USA) [11, 12]. Five used the validated ELISA kits provided by EIAAB (Catalogue number E1164H; Wuhan, China) to measure the levels of betatrophin [10, 14–16, 20]. The other two used ELISA kits provided by Cusabio (Human ANGPLT8 ELISA kit, CSB-EL028107HU; Cusabio) [13] and Aviscera Bioscience (SK00528-02, Aviscera Bioscience, Santa Clara, CA, USA) [22], respectively.

### 3.3. Overall Meta-Analysis

The overall level of circulating betatrophin in T2DM patients was higher than that in the nondiabetic controls with statistical significance (random-effect SMD 0.53; 95% CI, 0.13 to 0.94; *P* = 0.01). To be noted, significant statistical heterogeneity was observed among studies (*I* ^2^ = 86.1%, *P* < 0.001).

### 3.4. Subgroup Analysis of Body Mass in Participants

**Figure 2:**
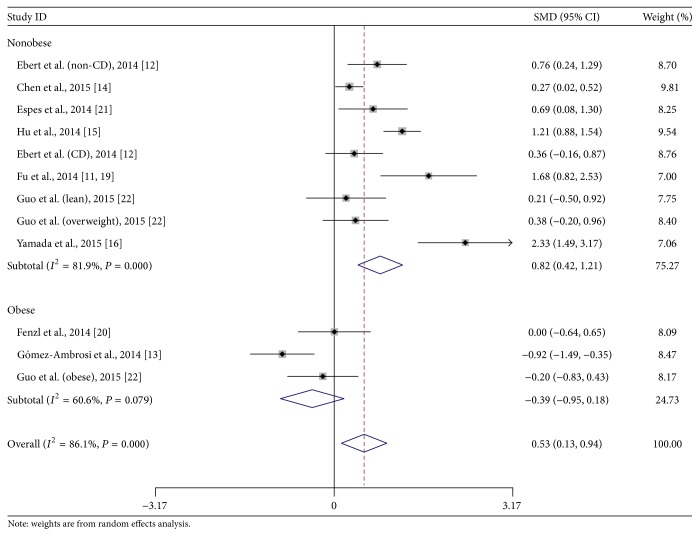
Subgroup analysis of circulating betatrophin level in T2DM or nondiabetic patients based on the body mass. CD: chronic hemodialysis; CI: confidential interval; SMD: standard mean difference. Test for subgroup differences: *χ* ^2^ = 11.6; df = 1; *P* = 0.0007; *I* ^2^ = 91.4%.

We introduced subgroup analysis based on whether the recruited participants were all obese. Three comparisons focused on obese population [13, 20, 22], while the other nine did not intentionally recruit patients and controls with obesity [10–12, 14–16]. In the subgroup of obesity, difference of the overall circulating betatrophin level between T2DM patients and nondiabetic adults did not reach statistical significance (random-effect SMD, −0.39; 95% CI, −0.95 to 0.18; *P* = 0.18; Figure 2). However, the overall betatrophin level in nonobese T2DM patients was much higher than that in the control group (random-effect SMD, 0.82; 95% CI 0.42 to 1.21; *P* < 0.001; Figure 2). The overall effect size was significantly different between two subgroups (*χ* ^2^ = 11.6, df = 1, *P* = 0.0007). Metaregression indicated that lower BMI in the T2DM group was associated with larger mean difference of serum betatrophin level between T2DM and nondiabetic adults (slope, −578.8; *t* = −2.7; *P* = 0.02, shown in Supplementary Figure 1 and Supplementary Table 4).

### 3.5. Subgroup Analysis of Betatrophin ELISA Kit

**Figure 3:**
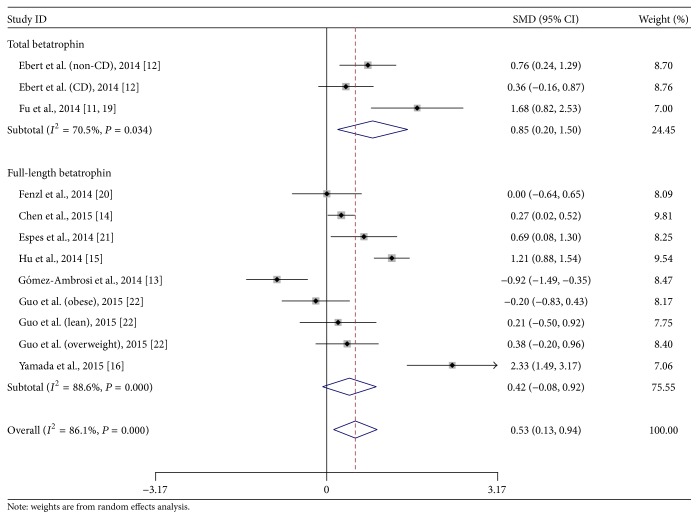
Subgroup analysis of circulating betatrophin level in T2DM or nondiabetic patients based on the ELISA kit selection. CD: chronic hemodialysis; CI: confidential interval; SMD: standard mean difference. Test for subgroup differences: *χ* ^2^ = 1.0; df = 1; *P* = 0.31; *I* ^2^ = 3.4%.

As circulating betatrophin level could be dominantly changed by the ELISA kit selection [19], we also introduced subgroup analysis based on the antibody design of the ELISA kits. Previous studies suggested that betatrophin could undergo protein cleavage to release the C-terminal fragment* in vivo*, the regulation of which was however unknown [19]. Antibodies of commercial ELISA kits were designed to act against either N-terminus or C-terminus of betatrophin. The antibody against N-terminus of betatrophin identified only full-length betatrophin, while the antibody against C-terminus identified total betatrophin including both full-length betatrophin and its C-terminal fragment [19]. According to the published literatures and E-mail contact with the production services, the ELISA kit from Phoenix identified C-terminus of betatrophin and detected total betatrophin, while all other kits were designed specifically for the full-length betatrophin. As shown in Figure 3, three comparisons in two studies [11, 12] detected total betatrophin and indicated that the total circulating betatrophin in T2DM patients was higher than that in the nondiabetic controls (SMD, 0.85; 95% CI, 0.20 to 1.50; *P* = 0.01). All the other nine comparisons detected only full-length betatrophin but did not find a statistical difference (SMD, 0.42; 95% CI, −0.08 to 0.92; *P* = 0.10). However, the trend of two subgroup results was similar without significant difference (*χ* ^2^ = 1.03, df = 1, and *P* = 0.31).

### 3.6. Correlation between Betatrophin and Metabolic Parameters

All included studies analyzed correlation between circulating betatrophin level and metabolic parameters, such as age, sex, body mass, blood pressure, glycemic parameters, lipid profiles, and renal and hepatic function (shown in Supplementary Table 5). All studies reported the correlation between betatrophin and fasting glucose, and five indicated a significant positive correlation [11, 12, 14–16], while one indicated a significant negative correlation [13]. Six out of nine included studies (with eight comparisons) reported the correlation between betatrophin and HbA1c, and three found a significant positive correlation [10, 15, 16]. Only two studies analyzed the correlation between betatrophin and postprandial glucose, and both showed a significant positive correlation [14, 15]. The results of parameters of insulin resistance, lipid profiles, and hepatic function were controversial among included studies.

### 3.7. Other Subgroup Analyses, Meta-Regression, Sensitivity Analysis, and Publication Bias Test

We also performed subgroup analyses according to lipid profiles, age, geographic region, diabetic duration, and antidiabetic medication usage, and the results were shown in Supplementary Table 6. Metaregression, along with these subgroup analyses, did not identify significantly associated factors of the mean difference of betatrophin, except BMI (shown in Supplementary Table 4). Remove-one sensitivity analysis was also conducted, and the pooled SMD was not altered by dropping any single comparison at a time (shown in Supplementary Figure 2). In addition, there was no evidence of publication bias (Begg's test, *P* = 0.58; Egger' test *P* = 0.81), and the funnel graph was shown in Supplementary Figure 3.

## 4. Discussion

This is the first meta-analysis of the association between circulating betatrophin and T2DM. Our study suggested that circulating betatrophin levels were significantly higher in nonobese T2DM patients, but not in obese ones, compared with those in nonobese nondiabetic adults.

Serving as a lipase activity regulator, betatrophin could induce postprandial triglyceride utility and storage in adiposity [23]. The name betatrophin was used since it was suggested by Yi and colleagues as a mediator of *β*-cell proliferation and a potential therapeutic target of diabetes [8]. However, several consequent studies indicated that betatrophin expression could be induced by high-fat diet and insulin, resulting in increased serum triglyceride and insulin resistance instead of improved glucose metabolism [9, 24, 25]. Knockout of betatrophin also failed to alter glucose profiles and *β*-cell mass in mice [4, 9]. However, current population-based studies indicated that betatrophin could be a biomarker candidate of diabetes and related disorders.

In the current meta-analysis, the overall difference of circulating betatrophin between the T2DM and nondiabetic population was statistically significant. But subgroup analysis indicated that betatrophin level was elevated only in nonobese T2DM patients instead of the obese ones. Metaregression also supported that BMI was an impact factor of betatrophin difference in T2DM patients. In the subgroup of obese participants, the overall circulating betatrophin level of obese T2DM patients was relatively lower than that in obese nondiabetic controls. However, the difference did not reach statistical significance. Only one study showed that the betatrophin level decreased significantly in obese diabetics, with the mean BMI of its population highest among all included studies (39.0 kg/m^2^ in obese T2DM group and 39.4 kg/m^2^ in obese nondiabetic group) [13]. Considering even higher betatrophin level was shown in the lean nondiabetic group in the same study, this significant change might suggest the association between a low circulating betatrophin level and T2DM with obesity, rather than the association between a high betatrophin level and obesity without T2DM. To be noted, the subgroup of obese population included only three studies with significant heterogeneity. The results from this subgroup should be treated with caution that potential confounding bias might be introduced. Further investigations of betatrophin level in overweight and obese population would be interesting for better understanding of the underlying pathogenesis.

To our knowledge, three reports directly compared the betatrophin level in lean and overweight/obese adults [11, 20, 22]. Only two of them investigated obese patients separately from the overweight ones [11, 22]. However, neither of these two studies showed significantly lower betatrophin in obese patients without diabetes, but higher level in overweight patients with or without T2DM. These results indicated that the association between circulating betatrophin and body mass was not linear, and the interactive effect of body mass in our meta-analysis appeared complicated. Thus, introduction of another variable might help. Animal experiments indicated that betatrophin was produced by liver and induced by insulin and food intake and supposed to be associated with lipid storage in mice [4, 23]. A recent study [26] indicated that recombinant irisin could induce the white adipocytes browning, body weight loss, and betatrophin elevation. Another human study confirmed the positive correlation between irisin and betatrophin [27]. It is interesting to introduce lipid storage in adipocytes in the analysis of the betatrophin level in both human and mice with diabetes in further investigation.

As shown in Supplementary Table 5, several parameters were suggested to be associated with circulating betatrophin in patients with and without diabetes, including age, sex, lipid profiles, insulin resistance, body mass, blood glucose, creatinine clearance, and hepatic function [10–16, 20, 22, 28, 29]. Unfortunately, most of these results were controversial and difficult to demonstrate the association. However, we noticed that the positive correlation between circulating betatrophin and blood glucose level, rather than parameters of insulin resistance or lipid profiles, was almost identical. Meanwhile, three studies from two centers investigated the association between circulating betatrophin and type 1 diabetes mellitus (T1DM) [16, 21, 27], and all three found a significant increased level of full-length betatrophin in T1DM. The BMI of the participants was low in all these three studies, and few of the T1DM patients were concomitant with dyslipidemia, insulin resistance, or other metabolic disorders [16, 21, 27]. These results suggested that betatrophin might critically participant in the glycemic metabolism in human-being. Although Gusarova and colleagues [9] did not confirm betatrophin as a regulator of beta cell proliferation, a recent study indicated a targeted delivery of betatrophin may induce beta-cell regeneration in a mice model [30]. The therapeutic potential of betatrophin in diabetes could not be ignored. But further well-designed animal experiments are required to derive more effective methodology of betatrophin overexpression before clinical trials.

As a liver-secreting adipokine, the association between betatrophin and liver enzyme was interesting in three of the included studies [13, 15, 22]. None of the three studies showed the significant correlation between betatrophin and ALT or AST. But two studies [13, 22] found significant correlation between betatrophin and *γ*-GT. Interestingly, betatrophin was indicated to be positively correlated with *γ*-GT in one study [22] but negatively correlated with *γ*-GT in the other [13]. Although the results were controversial, it indicated that liver metabolic status might regulate the serum level of betatrophin. Further investigation is required to demonstrate the association between circulating betatrophin in metabolic liver diseases.

Antiatherogenic treatment is another potential influential factor of circulating betatrophin but not well investigated in the current studies. Seven studies [10, 13–16, 20, 22] reported the therapeutic information of included patients, when four of them [13–15, 22] were free of ongoing antidiabetic treatment. Five [10, 14–16] reported the treatment of comorbidities. Espes and colleagues [10] introduced the only study analyzing the potential association between betatrophin and statin and metformin prescription, but they failed to find a difference with statistical significance. The trends of Espes et al.'s results could not be confirmed by the other six studies [13–16, 20, 22]. In detail, in Espes et al.'s study, patients treated with metformin appeared to have a higher level of betatrophin, while the betatrophin level in T2DM patients was similar with that in nondiabetic control in Fenzl et al.'s study (100% usage of metformin) [20]. In Espes et al.'s study, patients treated with statins appeared to have an equivalent or slightly lower level of betatrophin, while the betatrophin level difference between diabetic and nondiabetic patients was the highest in Yamada et al.'s study (46.7% statin usage in diabetic patients and none in nondiabetic controls) among all included studies [16]. It remained difficult to speculate the potential association between betatrophin level and exact antiatherogenic therapies. But it is very interesting to introduce further investigations.

Another important bias might be introduced by the ELISA kits of betatrophin. A previous report indicated that human betatrophin could be detected in the serum as either full-length form (detected by antibody against N-terminus) or C-terminal fragment (detected by antibody against C-terminus) [19]. However, our subgroup analysis did not show different results from studies using different antibodies. Although the analysis could be confounded by other parameters, it at least indicated that antibodies in the ELISA kits were unlikely to be the dominant factor influencing the circulating betatrophin concentration. Our subgroup analysis and metaregression also showed little influence of lipid profiles on the betatrophin difference between T2DM and nondiabetic controls.

Several limitations could not be omitted in our preliminary meta-analysis. First, all nine included studies were small in sample size with significantly heterogeneous population, which made our subgroup analysis and multivariable analysis in independent studies insufficient. Second, the measurement of betatrophin varies, and the absolute value of betatrophin was widely different between included studies. Third, as only three studies involving 166 participants were included in the subgroup of obese population, the lack of association between circulating betatrophin and T2DM in this subgroup required further confirmation by further investigations. Forth, few studies investigated the association between circulating betatrophin and antiatherogenic therapies, which can hardly be demonstrated based on current evidence. Fifty, the distribution of circulating betatrophin in most studies was not normally distributed within group with great variability. Sixth, only case-control studies were included in the meta-analysis, and the causation of betatrophin could not be concluded. Seventh, only circulating betatrophin level after overnight fasting was detected in T2DM patients. As a food intake-induced hormone, postprandial betatrophin after standard diet could be more meaningful in further investigation.

In summary, fasting circulating betatrophin level was associated with T2DM in nonobese patients. However, the physiology and metabolism of betatrophin remained unclear in human. Further population-based studies of betatrophin should be performed after strict stratification of potential confounding factors, especially body weight mass, lipid and glucose profiles, insulin resistance, laboratory measurement, and antiatherogenic therapy. And betatrophin might serve as a biomarker candidate of T2DM if all these variables were fully adjusted. Although results from animal experiments were controversial, it is still interesting to explore the potential role of betatrophin in diabetic prediction and therapeutics.

## Supplementary Material

This meta-analysis was prepared according to Meta-analyses of Observational Studies (MOOSE) guideline (supplementary table 1). The rationales of studies excluded during full-text reviewing were shown in the supplementary information. Besides meta-analysis, we performed meta-regression (supplementary figure 1), funnel plot (supplementary figure 2) and remove-on sensitivity analysis (supplementary figure 3) to explore potential biases of our meta-analysis. We also provided the detailed quality assessment scoring (supplementary table 2), prescriptive information of participants (supplementary table 3) and correlation results (supplementary table 5) of each included study. Non-significant results derived by meta-regression (supplementary table 4) and subgroup analyses (supplementary table 6) were also provided.

## Figures and Tables

**Table 1:** Characteristics of studies included in this meta-analysis.

Study ID	Country	Study design	Betatrophin (Elisa kit)	Case definition	Control source	Number^a^	Age, yrs^b^	Female, %^c^	BMI, kg/m^2^ ^d^	Baseline matching	NOS
Ebert et al. (non-CD), 2014 [12]	Germany	CCS	Total (Phoenix)	T2DM on ADT	Nondiabetic	30/30	63/63	46.7/63.3	29.1/28.2	NA	7

Ebert et al. (CD), 2014 [12]	Germany	CCS	Total (Phoenix)	T2DM on ADT and CD	Nondiabetic with eGFR >50 mL/min/1.73 m^2^	32/28	68/59	37.5/46.4	27.9/25.2	NA	7

Fu et al., 2014 [11, 19]	USA	CCS	Total (Phoenix)	T2DM	Nondiabetes	14/15	49.2/46.1	64.3/60.0	26.8/26.2	BMI, age	4

Fenzl et al., 2014 [20]	Austria	CCS	Full-length (EIAAB)	Obese T2DM on ADT	Obese nondiabetes	18/19	59.9/56.9	44.4/47.4	32.2/35.2	BMI, sex, and age	7

Chen et al., 2015 [14]	China	CCS	Full-length (EIAAB)	Newly diagnosed T2DM without ADT	NGT from REACTION study	112/137	60.7/60.2	48.2/54.7	23.4/23.0	BMI, sex, age, and blood lipid	8

Espes et al., 2014 [21]	Sweden	CCS	Full-length (EIAAB)	T2DM on ADT	Nondiabetes recruited through advertising	27/18	61.9/65.4	37.0/50.0	30.1/29.0	BMI, sex, and age	7

Hu et al., 2014 [15]	Spain	CCS	Full-length (EIAAB)	Newly diagnosed T2D without ADT	Healthy participants	83/83	48.4/48.0	37.3/34.9	25.1/25.3	Sex, age	6

Gómez-Ambrosi et al., 2014 [13]	Spain	CCS	Full-length (Cusabio)	Newly diagnosed obese T2DM without ADT	Obese NGT	15/75	49.2/47.3	53.3/49.3	39.0/39.4	NA	7

Yamada et al., 2015 [16]	Japan	CCS	Full-length (EIAAB)	T2DM on ADT	Healthy participants	30/12	56.0/55.6	50.0/41.7	25.7/22.7	Sex, age	5

Guo et al. (obese), 2015 [22]	China	CCS	Full-length (Aviscera Bioscience)	Obese T2DM	Obese NGT	19/20	45.4/40.8	42.1/50.0	33.5/33.0	NA	5

Guo et al. (overweight), 2015 [22]	China	CCS	Full-length (Aviscera Bioscience)	Overweight T2DM	Overweight NGT	23/23	50.7/49.4	56.5/56.5	28.0/27.1	NA	5

Guo et al. (lean), 2015 [22]	China	CCS	Full-length (Aviscera Bioscience)	Lean T2DM	Lean NGT	14/17	58.2/46.4	35.7/47.1	21.5/22.4	NA	5

ADT: antidiabetic treatment; BMI: body mass index; CCS: case-control study; CD: chronic hemodialysis; eGFR: evaluated glomerular filtration rate; NA: not available; NGT: normal glucose tolerance; NOS: the Newcastle-Ottawa Scale; T2DM: type 2 diabetes mellitus.

^a^The number of cases/number of controls.

^b^Mean age of cases/mean age of controls.

^c^Percentage of females in cases/percentage of females in controls.

^d^Mean BMI of cases/mean BMI of controls.

**Table 2:** Baseline characteristics of the enrolled studies.

Study ID	Fasting glucose, mmol/L	Fasting insulin, mIU/L	C-peptide, nmol/L	HOMA-IR	HbA1c, %	TC, mmol/L	TG, mmol/L	HDL-c, mmol/L	LDL-c, mmol/L
Ebert et al. (non-CD), 2014 [12]	7.60/5.10	6.88/6.48	NA	2.70/1.30	NA	4.90/5.30	1.40/1.10	1.20/1.40	2.90/3.50
Ebert et al. (CD), 2014 [12]	5.20/4.60	7.19/4.05	NA	1.40/0.80	NA	4.20/4.40	1.80/1.60	1.00/1.00	2.10/2.70
Fu et al., 2014 [11, 19]	11.17/5.19	NA	NA	NA	9.20/NA	4.62/4.56	2.26/1.64	1.17/1.35	2.83/2.54
Fenzl et al., 2014 [20]	7.29/5.19	10.06/10.08	1.09/1.02	3.72/2.72	7.72/5.73	5.48/3.95	1.96/1.37	1.33/1.73	3.22/2.55
Chen et al., 2015[14]	8.70/4.90	6.81/4.31	NA	3.09/1.07	7.70/5.50	5.00/4.90	1.50/1.40	1.40/1.50	2.90/2.80
Espes et al., 2014 [21]	8.50/6.20	NA	1.20/0.98	3.10/2.30	6.80/5.80	4.55/6.18	1.45/1.54	1.18/1.46	2.80/3.98
Hu et al., 2014 [15]	9.99/5.06	8.27/7.16	NA	NA	9.23/5.26	5.14/4.61	2.46/1.59	1.16/1.27	3.28/2.80
Gómez-Ambrosi et al., 2014 [13]	6.17/5.17	23.70/12.90	1.19/0.74	6.30/3.00	NA	5.22/5.04	2.39/1.21	1.37/1.40	2.77/3.08
Yamada et al., 2015 [16]	8.00/5.28	NA	NA	NA	8.8/5.2	5.40/5.15	2.16/1.90	1.16/1.42	3.39/3.28
Guo et al. (obese), 2015 [22]	7.41/5.16	21.00/16.58	2.84/3.08	6.91/3.93	7.59/5.56	4.95/5.16	2.10/1.89	0.96/1.24	3.01/3.22
Guo et al. (overweight), 2015 [22]	9.09/5.36	23.06/9.19	2.48/2.09	8.65/2.22	8.78/5.53	5.16/5.33	2.87/1.74	0.95/1.28	3.12/3.56
Guo et al. (lean), 2015 [22]	9.33/5.08	9.04/6.63	1.67/1.41	3.81/1.51	9.56/5.16	4.62/4.82	2.45/1.12	1.07/1.33	2.53/3.21

CD: chronic hemodialysis; HDL-c: high-density lipoprotein-cholesterol; HOMA-IR: homeostasis model assessment of insulin resistance; LDL-c: low-density lipoprotein-cholesterol; NA: not available; TC: total cholesterol; TG: triglyceride.

All data are presented as mean variables of cases/mean variables of controls.
