# Supplementary material for: Circulating Betatrophin in Patients with Type 2 Diabetes: A Meta-Analysis
Source: J Diabetes Res. 2015 Dec 1;2016:6194750. doi: 10.1155/2016/6194750 (PMC4678092; doi:10.1155/2016/6194750)
Supplement: Supplementary file 1 — This meta-analysis was prepared according to Meta-analyses of Observational Studies (MOOSE) guideline (supplementary table 1). The rationales of studies excluded during full-text reviewing were shown in the supplementary information. Besides meta-analysis, we performed meta-regression (supplementary figure 1), funnel plot (supplementary figure 2) and remove-on sensitivity analysis (supplementary figure 3) to explore potential biases of our meta-analysis. We also provided the detailed quality assessment scoring (supplementary table 2), prescriptive information of participants (supplementary table 3) and correlation results (supplementary table 5) of each included study. Non-significant results derived by meta-regression (supplementary table 4) and subgroup analyses (supplementary table 6) were also provided. [file 6194750.f1.docx]

**Supplementary information and legends.**

**Supplementary Information. Full-text examined articles and the reasons for exclusion.**

After examining the full-text of 27 articles, only nine ^[1-9]^ met our inclusion criteria. Three papers ^[10-12]^were excluded because the studied population was T1DM or obesity rather than T2DM. One paper was excluded for lacking of the control group ^[13]^. Fourteen papers ^[14-27]^ were excluded for the absence of original data reporting(such as reviews, editorials and comments).

**References**

[1] Ebert T, Kralisch S, Hoffmann A, Bachmann A, Lossner U, Kratzsch J, Bluher M, Stumvoll M, Tonjes A, Fasshauer M. Circulating angiopoietin-like protein 8 is independently associated with fasting plasma glucose and type 2 diabetes mellitus. J Clin Endocrinol Metab 2014, 99:E2510-7.

[2] Fu Z, Berhane F, Fite A, Seyoum B, Abou-Samra AB, Zhang R. Elevated circulating lipasin/betatrophin in human type 2 diabetes and obesity. Sci Rep 2014, 4:5013.

[3] Fenzl A, Itariu BK, Kosi L, Fritzer-Szekeres M, Kautzky-Willer A, Stulnig TM, Kiefer FW. Circulating betatrophin correlates with atherogenic lipid profiles but not with glucose and insulin levels in insulin-resistant individuals. Diabetologia 2014, 57:1204-8.

[4] Chen X, Lu P, He W, Zhang J, Liu L, Yang Y, Liu Z, Xie J, Shao S, Du T, Su X, Zhou X, Hu S, Yuan G, Zhang M, Zhang H, Liu L, Wang D, Yu X. Circulating betatrophin levels are increased in patients with type 2 diabetes and associated with insulin resistance. J Clin Endocrinol Metab 2015, 100:E96-E100.

[5] Espes D, Martinell M. Increased circulating betatrophin concentrations in patients with type 2 diabetes. Int J Endocrinol 2014, 2014:323407.

[6] Hu H, Sun W, Yu S, Hong X, Qian W, Tang B, Wang D, Yang L, Wang J, Mao C, Zhou L, Yuan G. Increased circulating levels of betatrophin in newly diagnosed type 2 diabetic patients. Diabetes care 2014, 37:2718-22.

[7] Gomez-Ambrosi J, Pascual E, Catalan V, Rodriguez A, Ramirez B, Silva C, Gil MJ, Salvador J, Fruhbeck G. Circulating betatrophin concentrations are decreased in human obesity and type 2 diabetes. J Clin Endocrinol Metab 2014, 99:E2004-9.

[8] Yamada H, Saito T, Aoki A, Asano T, Yoshida M, Ikoma A, Kusaka I, Toyoshima H, Kakei M, Ishikawa SE. Circulating betatrophin is elevated in patients with type 1 and type 2 diabetes. Endocrine J 2015.

[9] Guo K, Lu J, Yu H, Zhao F, Pan P, Zhang L, Chen H, Bao Y, Jia W. Serum betatrophin concentrations are significantly increased in overweight but not in obese or type 2 diabetic individuals. Obesity 2015.

[10] Wu S, Gao H, Ma Y, Fu L, Zhang C, Luo X. Characterisation of betatrophin concentrations in childhood and adolescent obesity and insulin resistance. Pediatr Diabetes 2014.

[11] Espes D, Lau J, Carlsson PO. Increased circulating levels of betatrophin in individuals with long-standing type 1 diabetes. Diabetologia 2014, 57:50-3.

[12] Espes D, Lau J, Carlsson PO. Increased levels of irisin in people with long-standing Type 1 diabetes. Diabetic medicine: a journal of the British Diabetic Association 2015.

[13] Tokumoto S, Hamamoto Y, Fujimoto K, Yamaguchi E, Okamura E, Honjo S, Ikeda H, Wada Y, Hamasaki A, Koshiyama H. Correlation of circulating betatrophin concentrations with insulin secretion capacity, evaluated by glucagon stimulation tests. Diabet Med 2015.

[14] Fu Z, Abou-Samra AB, Zhang R. An explanation for recent discrepancies in levels of human circulating betatrophin. Diabetologia 2014, 57:2232-4.

[15] Zhang R, Abou-Samra AB. A dual role of lipasin (betatrophin) in lipid metabolism and glucose homeostasis: consensus and controversy. Cardiovas diabetol 2014, 13:133.

[16] Kaestner KH. Betatrophin--promises fading and lessons learned. Cell Metabolism 2014, 20:932-3.

[17] Stewart AF. Betatrophin versus bitter-trophin and the elephant in the room: time for a new normal in beta-cell regeneration research. Diabetes 2014, 63:1198-9.

[18] Tseng YH, Yeh YH, Chen WJ, Lin KH. Emerging Regulation and Function of Betatrophin. Int J Mol Sci 2014, 15:23640-57.

[19] Levitsky LL, Ardestani G, Rhoads DB. Role of growth factors in control of pancreatic beta cell mass: focus on betatrophin. Cur opin pediatr 2014, 26:475-9.

[20] Ahnfelt-Ronne J, Madsen OD: Betatrophin. Islets 2014, 6:e28686.

[21] Li Y, Teng C. Angiopoietin-like proteins 3, 4 and 8: regulating lipid metabolism and providing new hope for metabolic syndrome. Int J Endocrinol 2014, 22:679-87.

[22] Lickert H. Betatrophin fuels beta cell proliferation: first step toward regenerative therapy? Cell Metabolism 2013, 18:5-6.

[23] Raghow R: Betatrophin. A liver-derived hormone for the pancreatic beta-cell proliferation. World J Diabetes 2013, 4:234-7.

[24] Seymour PA, Serup P. Bulking up on beta cells. New Eng J Med 2013, 369:777-9.

[25] Araujo TG, Oliveira AG, Saad MJ. Insulin-resistance-associated compensatory mechanisms of pancreatic Beta cells: a current opinion. Front Endocrinol 2013, 4:146.

[26] Crunkhorn S: Metabolic disorders. Betatrophin boosts beta-cells. Nat Rev Drug Discovery 2013, 12:504.

[27] Yi P, Park JS, Melton DA. Perspectives on the activities of ANGPTL8/betatrophin. Cell 2014, 159:467-8.

**Supplementary figure 1. Spot graph of meta-regression of body mass index (BMI) and circulating betatrophin.**


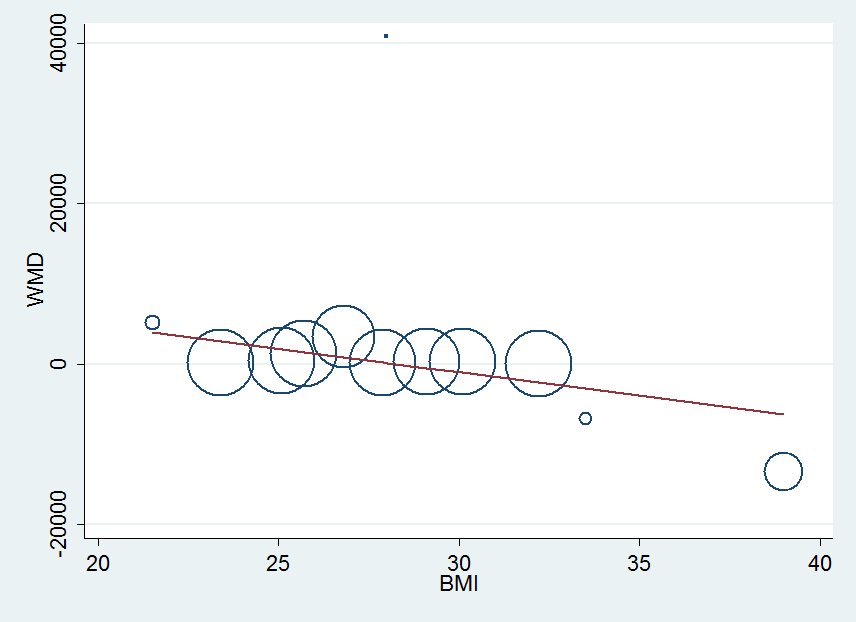


BMI: body mass index (kg/m^2^); WMD: weighted mean difference of betatrophin in each comparison (pg/mL).

**Supplementary figure 2. Begg's funnel plot of included studies.**



s.e.: standard error; SMD: standard mean deviation

**Supplementary figure 3. Remove-one sensitivity analysis of included studies.**





**Supplementary table 1. Checklist summarising compliance with MOOSE guidelines**

| **Item No** | **Recommendation** | **Reported on Page No** |
| --- | --- | --- |
| Reporting of background should include | | |
| 1 | Problem definition | 3 |
| 2 | Hypothesis statement | 3 |
| 3 | Description of study outcome(s) | 3 |
| 4 | Type of exposure or intervention used | 3 |
| 5 | Type of study designs used | 3 |
| 6 | Study population | 3 |
| Reporting of search strategy should include | | |
| 7 | Qualifications of searchers (eg, librarians and investigators) | 4 |
| 8 | Search strategy, including time period included in the synthesis and key words | 3 |
| 9 | Effort to include all available studies, including contact with authors | 3 |
| 10 | Databases and registries searched | 3 |
| 11 | Search software used, name and version, including special features used (eg, explosion) | 3 |
| 12 | Use of hand searching (eg, reference lists of obtained articles) | 3 |
| 13 | List of citations located and those excluded, including justification | 5 |
| 14 | Method of addressing articles published in languages other than English | 3-4 |
| 15 | Method of handling abstracts and unpublished studies | 3-4 |
| 16 | Description of any contact with authors | NA |
| Reporting of methods should include | | |
| 17 | Description of relevance or appropriateness of studies assembled for assessing the hypothesis to be tested | 3 |
| 18 | Rationale for the selection and coding of data (eg, sound clinical principles or convenience) | 3 |
| 19 | Documentation of how data were classified and coded (eg, multiple raters, blinding and interrater reliability) | 4 |
| 20 | Assessment of confounding (eg, comparability of cases and controls in studies where appropriate) | 4 |
| 21 | Assessment of study quality, including blinding of quality assessors, stratification or regression on possible predictors of study results | 4 |
| 22 | Assessment of heterogeneity | 4 |
| 23 | Description of statistical methods (eg, complete description of fixed or random effects models, justification of whether the chosen models account for predictors of study results, dose-response models, or cumulative meta-analysis) in sufficient detail to be replicated | 4 |
| 24 | Provision of appropriate tables and graphics | 5 |
| Reporting of results should include | | |
| 25 | Graphic summarizing individual study estimates and overall estimate | 5-6 |
| 26 | Table giving descriptive information for each study included | 5 |
| 27 | Results of sensitivity testing (eg, subgroup analysis) | 6-7 |
| 28 | Indication of statistical uncertainty of findings | 6-7 |
| Reporting of discussion should include | | |
| 29 | Quantitative assessment of bias (eg, publication bias) | 7 |
| 30 | Justification for exclusion (eg, exclusion of non-English language citations) | 5 |
| 31 | Assessment of quality of included studies | 5 |
| Reporting of conclusions should include | | |
| 32 | Consideration of alternative explanations for obsercved results | 7-10 |
| 33 | Generalization of the conclusions (ie, appropriate for the data presented and within the domain of the literature review) | 10 |
| 34 | Guidelines for future research | 10 |
| 35 | Disclosure of funding source | 10 |

*From*: Stroup DF, Berlin JA, Morton SC, et al, for the Meta-analysis Of Observational Studies in Epidemiology (MOOSE) Group. Meta-analysis of Observational Studies in Epidemiology. A Proposal for Reporting. *JAMA*. 2000;283(15):2008-2012. doi: 10.1001/jama.283.15.2008.

**Supplementary table 2. Newcastle-Ottawa Quality Assessment Scoring of each included studies**

| Study ID | Selection | Comparability | Exposure | Total |
| --- | --- | --- | --- | --- |
| Ebert, 2014 | ★★★/★★★★ | ★★/★★ | ★★/★★★ | 7 |
| Ambrosi, 2014 | ★★★/★★★★ | ★★/★★ | ★★/★★★ | 7 |
| Fenzl, 2014 | ★★★/★★★★ | ★★/★★ | ★★/★★★ | 7 |
| Chen, 2014 | ★★★★/★★★★ | ★★/★★ | ★★/★★★ | 8 |
| Fu, 2014 | -/★★★★ | ★★/★★ | ★★/★★★ | 4 |
| Espes, 2014 | ★★★/★★★★ | ★★/★★ | ★★/★★★ | 7 |
| Hu, 2014 | ★★/★★★★ | ★★/★★ | ★★/★★★ | 6 |
| Guo, 2015 | ★/★★★★ | ★★/★★ | ★★/★★★ | 5 |
| Yamada, 2015 | ★/★★★★ | ★★/★★ | ★★/★★★ | 5 |

Selection: maximum of 4 stars (adequacy of case definition; representativeness of the cases; selection of controls; and adequacy of control definition).

Comparability: maximum of 2 stars (comparability between cases and controls).

Exposure: maximum of 3 stars (adequacy of exposure, non-response rate).

**Supplementary table 3. Summary of prescriptive information of participants in each included studies.**

| Study ID | Antidiabetic medication | | Lipid-lowering medication | Antihypertensive medication |
| --- | --- | --- | --- | --- |
|  | OAD | insulin |  |  |
| Ebert (non-CD), 2014 | NR | NR | NR | NR |
| Ebert (CD), 2014 | NR | NR | NR | NR |
| Fu , 2014 | NR | NR | NR | NR |
| Fenzl, 2014 | 100%# | 0 | NR | NR |
| Chen, 2014 | 0 | 0 | 0 | 20% |
| Espes, 2014 | 92.5%# | 11.1% | 48%/22%* | 63%/28%* |
| Hu, 2014 | 0 | 0 | 0 | 0 |
| Ambrosi, 2014 | 0 | 0 | NR | NR |
| Yamada, 2015 | 100%# | 0 | 46.7%/0* | NR |
| Guo (obese), 2015 | NR | NR | 0 | 0 |
| Guo (overweight), 2015 | NR | NR | 0 | 0 |
| Guo (lean), 2015 | NR | NR | 0 | 0 |

NR = not reported, OAD = oral antidiabetic drugs

* Indicates data is presented as percentage of cases/ variables of controls

# In Fenzl's study, 13 out of 18 patients with T2DM received metformin monotherapy, while the other five received combination therapy of metformin and sulfonylurea. In Espes's study, 19 out of 27 T2DM patients received metformin as monotherapy. Three received combination therapy of metformin and another OAD (detail not reported). Two received non-metformin OAD monotherapy (detail not reported). One received combination therapy of metformin and insulin. In Yamada's study, all 30 T2DM patients received OAD, but the detailed strategy was not reported.

**Supplementary table 4. Summary of meta-regression of circulating betatrophin.**

| Covariates | Number of comparisons | Slope [95% CI] | t | P |
| --- | --- | --- | --- | --- |
| TC | 12 | -1862.8 [-8487.3, 4761.6] | -0.6 | 0.55 |
| TG | 12 | -1216.0 [-8225.0, 5793.1] | -0.4 | 0.71 |
| HDL-c | 12 | -10656.9 [-31218.9, 9905.2] | -1.2 | 0.28 |
| LDL-c | 12 | 1041.1 [-6350.9, 8433.1] | 0.3 | 0.76 |
| FBG | 12 | 739.5 [-384.5, 1863.5] | 1.5 | 0.17 |
| BMI | 12 | -578.8 [-1057.5, -100.1] | -2.7 | 0.02 |

BMI: body mass index; CI: confidential interval; FBG: fasting blood glucose; HDL-c: high-density lipoprotein cholesterol; LDL-c: low density lipoprotein-cholesterol; TC: total cholesterol; TG: triglyceride.

**Supplementary table 5. Summary of correlations between betatrophin and metabolic parameters in each included studies**

| Study ID | Age | Sex | BMI | WHR | SBP | DBP | HbA_1c_ | FPG | 2h-glucose | C-peptide | FINS | HOMA-IR | TC | TG | HDL-c | LDL-c | FFA |
| --- | --- | --- | --- | --- | --- | --- | --- | --- | --- | --- | --- | --- | --- | --- | --- | --- | --- |
| Ebert, 2014 (#) | -0.21* | - | 0.25* | -0.18 | 0.02 | 0.13 | - | 0.29* | - | - | 0.18* | 0.26* | 0.11 | -0.01 | 0.30* | -0.02 | 0.06 |
| Fu, 2014 (#) | - | - |  | - | - | - | - | 0.42* | - | - | 0.36* | - | NS | NS | NS | NS | - |
| Fenzl, 2014 (T2DM) | 0.09 | - | -0.23 | - | - | - | 0.18 | 0.04 | - | 0.22 | -0.04 | 0.02 | 0.55* | 0.06 | 0.20 | 0.61* | - |
| Chen, 2014 (T2DM) | 0.43* | - | -0.15 | - | - | - | 0.06#& | 0.11*#& | 0.12*#& | - | 0.07#& | 0.11*#& | -0.26* | - | -0.23* | -0.26* | - |
| Espes, 2014 (T2DM) | 0.08 | - | 0.15 | -0.42 | - | - | 0.48* | 0.08 | - | 0.10 | - | 0.11 | -0.06 | -0.12 | -0.02 | 0.09 | - |
| Hu, 2014 (T2DM) | 0.32* | - | 0.16 | 0.02& | 0.07& | 0.03& | 0.30*& | 0.35*& | 0.36*& | - | 0.27*& | - | 0.07& | 0.04& | -0.03& | 0.03& | - |
| Ambrosi, 2014 (#&) | 0.21* | 0.48* | -0.40* | - | -0.31* | -0.30* | - | -0.22* | - | -0.36* | -0.30* | -0.30* | -0.12 | -0.33* | 0.51* | -0.19* | - |
| Yamada, 2015 (#) | 0.36* | - | 0.14 | - | - | - | 0.55* | 0.46* | - | 0.32* | - | - | 0.17 | 0.40* | -0.25* | 0.12 | - |
| Guo, 2015 (T2DM) | -0.13 | - | 0.02 | 0.03 | 0.10 | 0.21 | -0.02 | 0.07 | - | 0.08 | 0.26 | 0.29* | 0.12 | 0.01 | 0.02 | 0.10 | - |

(continued)

| Study ID | Creatinine | eGFR | ALT | AST | γ-GT | Leptin | hsIL-6 | Uric acid |
| --- | --- | --- | --- | --- | --- | --- | --- | --- |
| Ebert, 2014 (#) | -0.23* | 0.19* | - | - | - | 0.22* | -0.18* | - |
| Fu, 2014 (#) | - | - |  | - | - | - | - | - |
| Fenzl, 2014 (T2DM) | - | - | - | - | - | - | - | - |
| Chen, 2014 (T2DM) | - | - | - | - | - | - | - | - |
| Espes, 2014 (T2DM) | 0.34 | -0.21 | - | - | - | - | - | - |
| Hu, 2014 (T2DM) | - | - | 0.12& | 0.15& | 0.05& | - | - | - |
| Ambrosi, 2014 (#&) | -0.37* | - | -0.01 | - | -0.29* | 0.00 | - | -0.49* |
| Yamada, 2015 (#) | - | -0.17 | - | - | - | - | - | - |
| Guo, 2015 (T2DM) | - | - | 0.23 | 0.21 | 0.31* | - |  | - |

ALT = alanine aminotransferase, AST = aspartate aminotransferase, BMI = body mass index, DBP =diastolic blood pressure, eGFR = estimated glomerular filtration rate, FFA = free fatty acid, FINS = fasting insulin, FPG = fasting plasma glucose, γ-GT = gamma-glutamyltransferase, HbA_1c_ = hemoglobin A_1c_, HDL-c = high-density lipoprotein-cholesterol, HOMA-IR = homeostasis model assessment of insulin resistance, hsIL-6 = high-sensitivity interleukin-6, LDL-c = low-density lipoprotein-cholesterol, NS = not significant (*P*>0.05, without providing specific r and *P* value), SBP = systolic blood pressure, TC = total cholesterol, T2DM = type 2 diabetes mellitus, TG = triglyceride, WHR = waist-to-hip ratio.

* *P* <0.05;

# Indicates data of all participants;

& Indicates adjusted correlation.

**Supplementary table 6. Subgroup analysis of circulating betatrophin level.**

| **Subgroup titles** | **No. of comparisons*** | **No. of participants** | **Statistical methods** | **OR [95% CI]** |
| --- | --- | --- | --- | --- |
| **1 Continent** | 12 | 894 | SMD (Random, 95% CI) | 0.53 [0.13, 0.94] |
| 1.1 Europe | 5 | 292 | SMD (Random, 95% CI) | 0.18 [-0.42, 0.78] |
| 1.2 North America | 1 | 29 | SMD (Random, 95% CI) | 1.68 [0.82, 2.53] |
| 1.3 Asia | 6 | 573 | SMD (Random, 95% CI) | 0.67 [0.09, 1.24] |
| **2 Disease duration**  2.1 previously diagnosed T2DM  2.2 newly diagnosed T2DM  2.3 non-specific T2DM  **3 Treatment**  3.1 under treatment  3.2 not clear  3.3 without treatment  **4 Obesity**  4.1 non-obesity  4.2 obesity  **5 Age**  5.1 60y or more  5.2 under 60y  6 **TG**  6.1 lower half of mean serum TG  6.2 higher half of mean serum TG  **7 LDL-c**  7.1 lower half of mean serum LDL-c  7.2 higher half of mean serum LDL-c  8 **Betatrophin** **measurement**  8.1 total betatrophin  8.2 full-length betatrophin | 12  8  3  1  12  5  1  6  12  9  3  12  4  8  12  6  6  12  6  6  12  3  9 | 894  360  505  29  894  244  29  621  894  728  166  894  414  480  894  490  404  894  315  579  894  149  745 | SMD (Random, 95% CI)  SMD (Random, 95% CI)  SMD (Random, 95% CI)  SMD (Random, 95% CI)  SMD (Random, 95% CI)  SMD (Random, 95% CI)  SMD (Random, 95% CI)  SMD (Random, 95% CI)  SMD (Random, 95% CI)  SMD (Random, 95% CI)  SMD (Random, 95% CI)  SMD (Random, 95% CI)  SMD (Random, 95% CI)  SMD (Random, 95% CI)  SMD (Random, 95% CI)  SMD (Random, 95% CI)  SMD (Random, 95% CI)  SMD (Random, 95% CI)  SMD (Random, 95% CI)  SMD (Random, 95% CI)  SMD (Random, 95% CI)  SMD (Random, 95% CI)  SMD (Random, 95% CI) | 0.53 [0.13, 0.94]  0.53 [0.10, 0.97]  0.21 [-0.76, 1.18]  1.68 [0.82, 2.53]  0.53 [0.13, 0.94]  0.78 [0.16, 1.40]  1.68 [0.82, 2.53]  0.18 [-0.39, 0.76]  0.53 [0.13, 0.94]  0.82 [0.42, 1.21]  -0.39 [-0.95, 0.18]  0.53 [0.13, 0.94]  0.43 [0.19, 0.67]  0.56 [-0.13, 1.25]  0.53 [0.13, 0.94]  0.33 [0.07, 0.58]  0.79 [-0.07, 1.64]  0.53 [0.13, 0.94]  0.43 [-0.20, 1.07]  0.63 [0.05, 1.21]  0.53 [0.13, 0.94]  0.85 [0.20, 1.50]  0.42 [-0.08, 0.92] |

CI: confidential interval; LDL-c: low density lipoprotein-cholesterol; OR: odds ratio; SMD: standard mean deviation; T2DM: type 2 diabetes mellitus; TG: triglyceride.

* Nine studies with twelve comparisons were enrolled, and five different results based on five different groups were derived from two studies.
